# Novel Afro-Caribbean Prostate Cancer Model Reveals Ancestry-Specific Drug Vulnerabilities with Therapeutic Implications for Black Patients

**DOI:** 10.1158/2767-9764.CRC-25-0254

**Published:** 2025-10-03

**Authors:** Simone Badal, Bor-Jang Hwang, Ashlianne Nelson, Kristoff Frank, Tanisha Maitre, Magdalene Nwokocha, Rory Thompson, Belinda Morison, Rajini Haraksingh, Valerie Odero-Marah, Camille Ragin

**Affiliations:** 1 Department of Basic Medical Sciences, Faculty of Medical Sciences Teaching and Research Complex, The University of the West Indies, Mona, Jamaica.; 2 African Caribbean Cancer Consortium, Philadelphia, Pennsylvania.; 3 Center for Health Disparities Research and Innovation, Department of Biology, Morgan State University, Baltimore, Maryland.; 4 Cancer Prevention and Control Program, Fox Chase Cancer Center, Philadelphia, Pennsylvania.; 5 Department of Pathology, University Hospital of the West Indies, Mona, Jamaica.; 6 Section of Surgery, Department of Surgery, Radiology, Anaesthesia and Intensive Care, Faculty of Medical Sciences, The University of the West Indies, Mona, Jamaica.; 7 Department of Life Sciences, Faculty of Science and Technology, The University of the West Indies, St. Augustine, Trinidad and Tobago.

## Abstract

In the era of targeted therapeutics, the inadequate representation of Black populations in prostate cancer models limits effective drug screening. In this study, we introduce ACRJ-PC28, a novel Afro-Caribbean prostate cancer cell line, and evaluate its responses to five anticancer drugs (docetaxel, cabazitaxel, abiraterone, olaparib, and enzalutamide) and betaine. We compare these responses with those of established prostate cancer cell lines from Black (MDA-PCA-2b) and White (DU-145 and PC-3) donors using three distinct viability assays. We observed ancestry-dependent drug sensitivities: abiraterone showed remarkable selectivity for ACRJ-PC28 (IC_50_ = 1.10 μmol/L), being 4.6- to 13.1-fold more potent than in other cell lines, whereas enzalutamide demonstrated pronounced racial differences, being 3 to 5 times less effective in cell lines from Black donors (IC_50_ = 206 μmol/L for ACRJ-PC28; 104 μmol/L for MDA-PCA-2b) versus cell lines from White donors (IC_50_ = 37 μmol/L for PC-3; 48 μmol/L for DU-145). RNA sequencing analysis revealed consistent underexpression of TNF family genes, particularly *TNFRSF14*, in prostate cancer cells from Black donors correlating with differential drug responses. Despite underexpressing *AR*, the ACRJ-PC28 line exhibited exceptional sensitivity to abiraterone, consistent with clinical observations that Black patients with prostate cancer respond better to this therapy. This aligns with its neuroendocrine phenotype in the source patient, who succumbed within 1 year despite androgen deprivation therapy. Our findings suggest that incorporating diverse prostate cancer models in preclinical screening could guide personalized treatment strategies for Black patients who experience disproportionate prostate cancer mortality by identifying ancestry-specific drug vulnerabilities that inform optimal therapeutic combinations.

**Significance:**

This study introduces ACRJ-PC28, the first Afro-Caribbean prostate cancer cell line, revealing ancestry-dependent drug sensitivities that could explain differential clinical outcomes. The findings demonstrate critical gaps in current preclinical models and support incorporating diverse cell lines to develop personalized treatment strategies for underrepresented populations experiencing disproportionate cancer mortality.

## Introduction

The treatment of cancers primarily through radical surgeries and cauterization dates back to a millennium (1). The ineffectiveness of both processes led to further investigation, correcting the notion that tumors were immobile. This realization underscored the limitations of surgery and cauterization alone, as cancer cells could be deposited on organs other than their original site. Subsequently, radiotherapy, developed in the early 1900s, became a common treatment alongside surgery, followed by the introduction of chemotherapy in the 1940s (2). Among the various cancer types in which these translational challenges are evident, prostate cancer represents a particularly compelling case study. Not only is it one of the most common malignancies in men worldwide, but it also exhibits striking disparities in incidence and outcomes across different populations. The evolution of prostate cancer therapies has followed the broader historic trajectory of oncology treatment development—from surgical approaches to radiation, chemotherapy, and now targeted molecular interventions. However, the effectiveness of these advances has varied considerably across different patient populations, making prostate cancer an ideal model for investigating how preclinical research tools might better reflect and address real-world clinical heterogeneity.

Efforts continue to search for lead molecules that can effectively target cancer cells; however, this approach remains in progress. Initially, the primary models used, which are still relevant, were mice. During the early 1900s, alongside the use of mice, the development of cell lines aimed to address the many challenges encountered with *in vivo* models. Cell lines provide a controlled environment for identifying specific intracellular and extracellular targets and their direct manipulation, ultimately proving cost-effective. They are believed to serve as a bridge between basic science and clinical applications in cancer treatment (3). Despite both *in vitro* and *in vivo* models remaining as predominant preclinical models, a significant challenge over the years has been the poor translatability of these models to clinical applications, referred to as the journey “from bench to bedside” (4).

This research specifically focuses on prostate cancer, a disease that has shown a decline in mortality rates over the years, leading to improved survival and quality of life across many populations (5). However, these improvements have not been equitably distributed, with significantly better outcomes observed among White men (6). Despite advances in treatment, Black men continue to experience 1.5 to 2 times higher incidence and mortality rates (7), with this disparity particularly pronounced in regions such as the Caribbean and Africa (8). Whereas socioeconomic factors contribute to these disparities, mounting evidence suggests that biological differences in tumor biology and treatment response may play critical roles, yet these mechanisms remain understudied due to limited diversity in available preclinical models.

Despite the clear evidence of racial disparities in prostate cancer outcomes, there remains a critical gap in our understanding of the biological mechanisms underlying these differences. This gap is perpetuated by the lack of diversity in established cell line models, with fewer than 10% of commercially available prostate cancer cell lines derived from Black patients and none from Caribbean populations (9, 10). This inadequate representation limits our ability to identify ancestry-specific therapeutic vulnerabilities and develop targeted treatment approaches. Given that prostate cancer in Black men often presents with unique molecular features and more aggressive phenotypes, developing and characterizing cell lines that reflect this population’s genetic ancestry is essential for advancing personalized treatment strategies.

Androgen deprivation therapy (ADT) with or without chemotherapy is the standard recommendation for men with metastatic prostate cancer. Chemotherapy is especially utilized for aggressive forms of prostate cancer such as neuroendocrine prostate cancer (11) which disproportionately affects Black men. The anticancer industry has witnessed the evolution of treatments for metastatic prostate cancer, beginning with the antineoplastic chemotherapy drug mitoxantrone in 1996, followed by docetaxel and cabazitaxel in 2004 and 2010, respectively (12, 13). More recently, targeted approaches including androgen synthesis inhibitors (abiraterone), androgen receptor (AR) signaling inhibitors (enzalutamide), immunotherapies (pembrolizumab), and PARP inhibitors (olaparib) have entered clinical practice (14–17). However, most clinical trials leading to these approvals have enrolled predominantly White participants, with Black patients representing less than 10% of study populations. This underrepresentation raises questions about the generalizability of efficacy and safety findings across diverse populations, particularly given emerging evidence of ancestry-dependent drug responses.

Cell viability assessment is critical for accurately determining drug efficacy, yet reliance on a single-assay method can lead to misleading conclusions due to cell-specific metabolic differences. Traditional approaches like MTT/MTS assays that measure mitochondrial activity, neutral red that assesses lysosomal integrity, and crystal violet that quantifies adherent cells each have inherent limitations when used alone, particularly when comparing cell lines with different growth characteristics and metabolic profiles. In our study, we used a multiple-assay approach to determine IC_50_ values, capturing complementary aspects of cellular response to treatment. This comprehensive strategy was especially important given the observed differences in growth patterns and metabolic activities between prostate cancer cell lines of different racial backgrounds, allowing for more reliable drug response comparisons and potentially revealing ancestry-specific therapeutic vulnerabilities that might be missed using conventional single-assay methods.

In our study, we address this critical research gap by developing and characterizing ACRJ-PC28, a novel cell line derived from an Afro-Caribbean patient with aggressive prostate cancer. We adopted a unique approach to determine the IC_50_ values of cytotoxic agents using multiple cell viability assays rather than conventional single-assay methods. This approach was necessitated by our observation of variations in growth patterns among prostate cancer cell lines from different racial backgrounds and their diverse responses to different assays. By comparing responses of Black and White prostate cancer cell lines to a panel of clinically relevant anticancer drugs, we aimed to identify potential ancestry-specific therapeutic vulnerabilities that could inform more personalized treatment strategies for Black patients with prostate cancer, particularly those of Caribbean descent, who face some of the highest mortality rates globally.

## Materials and Methods

This study used a multipronged experimental approach to evaluate differential drug responses in prostate cancer cell lines derived from patients of diverse racial backgrounds. Our methodology centered on comparing the cytotoxic effects of five clinically relevant anticancer drugs (docetaxel, cabazitaxel, abiraterone, enzalutamide, and olaparib) and one natural product, betaine, across four prostate cancer cell lines (DU-145, PC-3, MDA-PCA-2b, and ACRJ-PC28) using multiple complementary viability assays [CellTiter AQueous (CTA), neutral red, and crystal violet assays] to ensure robust measurement of drug sensitivity. We conducted comprehensive RNA sequencing (RNA-seq) analysis to identify potential molecular mechanisms underlying observed differences in drug response. This research was conducted through collaboration between the AntiCancer Research Jamaica Laboratory, University of the West Indies Mona, Morgan State University, and Fox Chase Cancer Center. Data collection spanned 15 months, with repeated testing performed 2 to 3 times to ensure the validity and reproducibility of the findings.

Culture medium: DMEM (Corning Cellgro, #MT10-013-CV; RRID: SCR_013959), RPMI-1640 (Corning Cellgro, #MT10-040-CV; RRID: SCR_013959), mouse EGF, and penicillin/streptomycin were purchased from Corning Cellgro. FBS (Fisher Scientific, #35-010-CV; RRID: SCR_008452), human recombinant insulin, and 10× PBS were purchased from Fisher Scientific (RRID: SCR_008452). Keratinocyte Growth Kit (ATCC, PCS-200-040; RRID: SCR_001672) and F-12K Medium (ATCC, #30-2004; RRID: SCR_001672) were obtained from the ATCC. The CellTiter 96 AQueous One Solution Cell Proliferation Assay (MTS, Promega, #G3581; RRID: SCR_006724) was purchased from Promega. Abiraterone (MedChemExpress, #HY-70013; RRID: SCR_021293), cabazitaxel (MedChemExpress, #HY-15459; RRID: SCR_021293), enzalutamide (MedChemExpress, #HY-70002; RRID: SCR_021293), and olaparib (MedChemExpress, #HY-10162; RRID: SCR_021293) were purchased from MedChemExpress (RRID: SCR_021293).

Commercial cell lines DU-145 (RRID: CVCL_0105), PC-3 (RRID: CVCL_0035), and MDA-PCA-2b (RRID: CVCL_4748) were purchased directly from the ATCC (RRID: SCR_001672) with authentication certificates and *Mycoplasma*-free certification. The ACRJ-PC28 cell line was authenticated using short tandem repeat profiling at IDEXX BioAnalytics in November 2021. Short tandem repeat profiles were compared against ATCC, DSMZ, and other reference databases to confirm cell line identity and absence of cross-contamination (18). All cell lines were routinely tested for *Mycoplasma* contamination using PCR-based MycoAlert Mycoplasma Detection Kit (Lonza; RRID: AB_2665610) every 3 months during the experimental period (January 2022–April 2023) and confirmed to be negative. Cell lines were maintained within 10 passages from receipt or authentication to minimize genetic drift and phenotypic changes during experiments. Genetic ancestry of the ACRJ-PC28 cell line was confirmed through admixture analysis using YRI (Yoruba in Ibadan, Nigeria) and CEU (Utah residents with Northern European and Western European ancestries) populations from the 1000 Genomes Project as reference, which indicated 97% African ancestry and confirmed male origin (18).

### Cell culture

PC-3 cells derived from the bone metastasis of a 63-year-old White male with grade IV prostatic adenocarcinoma were obtained from the ATCC and maintained in RPMI-1640 supplemented with 100 IU penicillin, 100 μg/mL streptomycin, and 10% FBS. DU-145 cells derived from the brain of a 69-year-old White male with prostate cancer were obtained from the ATCC and maintained in DMEM supplemented with 100 IU penicillin, 100 μg/mL streptomycin, and 10% FBS. MDA-PCA-2b derived from the bone metastasis of a 63-year-old Black male with androgen-independent prostate adenocarcinoma was obtained from the ATCC, and cells were grown in F-12K Medium supplemented with 20% non–heat-inactivated FBS, 25 ng/mL cholera toxin, 10 ng/mL mouse EGF, 0.005 mmol/L phosphoethanolamine, 100 pg/mL hydrocortisone, 45 nmol/L sodium selenite, 0.005 mg/mL human recombinant insulin, 100 IU penicillin, and 100 μg/mL streptomycin. ACRJ-PC28 cells derived from a 69-year-old Jamaican Black male with prostate adenocarcinoma were cultured in Keratinocyte Serum-Free Medium supplemented with ATCC Keratinocyte Growth Kit (PCS-200-040). The final concentration for each component was as follows: bovine pituitary extract (0.4%), recombinant human TGFα (0.5 ng/mL), hydrocortisone (100 ng/mL), insulin (5 μg/mL), epinephrine (1.0 μmol/L), apo-transferrin (5 μg/mL), EGF (5 ng/mL), penicillin (100 IU), and streptomycin (100 μg/mL).

### Drug treatments

Because the cell growth rate and conditions varied, to achieve the best detection confluency, prostate cancer cells were seeded in 96-well flat-bottom tissue culture plates at the following cell densities: 1,000 cells/well for PC-3, 2,000 cells/well for DU-145, 10,000 cells/well for MDA-PCA-2b, and 3,000 cells/well for ACRJ-PC28. One day after seeding, the cells were treated with the indicated drug concentrations (Table 1) for 3 days. To ensure uniform solvent quantities in each well, abiraterone, cabazitaxel, docetaxel, enzalutamide, and olaparib were dissolved in DMSO. As such, 500× drug concentrations were prepared in a 1:1 serial dilution in DMSO immediately before drug treatment.

**Table 1.** Drug concentration ranges used for cytotoxicity testing. Concentration ranges were determined based on literature review of previously published studies (19–25) for each compound. Drug concentrations are shown in the units indicated for each column header.

Abiraterone (mmol/L)	Betaine (mmol/L)	Cabazitaxel (nmol/L)	Docetaxel (nmol/L)	Enzalutamide (mmol/L)	Olaparib (mmol/L)
0	0	0	0	0	0
0.625	0.78125	0.01	0.01	0.625	0.15625
1.25	1.5625	0.025	0.025	1.25	0.3125
2.5	3.125	0.05	0.05	2.5	0.625
5	6.25	0.075	0.075	5	1.25
10	12.5	0.1	0.1	10	2.5
15	25	0.15	0.15	20	5
20	50	0.3	0.3	40	10
25	100	0.5	0.5	80	20
30	200	1	1	100	40
35	400	2	2	160	80
40	600	5	5	200	100
​	800	10	10	​	160
​	1,000	15	15	​	200

Subsequently, a consistent 1:500 dilution was used to establish the final treatment concentrations for PC-3 and DU-145 cells. Given the poor adherence of MDA-PCA-2b cells and the nature of ACRJ-PC28 cells (which are sensitive to disturbances), the original culture medium was not replaced with drug treatment media. Instead, 10× drug concentrations were prepared, and 11 μL of each concentrated treatment were added to the original 100 μL to achieve the specified concentration levels (as outlined in Table 1). A 5 mol/L betaine solution was initially prepared in PBS as a stock, and 10× treated betaine solutions were freshly prepared, with 1/10th of the final volume subsequently added to each well. Cell images were captured using a Moticam 400 microscopic imaging system 2 days after treatment, and viability assays were conducted 3 days after the drug treatments.

### Cell viability assays

For the MTS Cell Proliferation Assay (26), 20 μL per well of MTS (CTA) reagent was added per well, and the plates incubated for 2 hours before measuring the absorbance at 490 nm using a BioTek plate reader (Agilent Technologies). For neutral red staining (27), the plates were incubated for 2 hours in the respective medium for each cell line containing 40 μg/mL of neutral red (3-amino-7-dimethylamino-2-methyl-phenazine hydrochloride). To address the poor attachment of MDA-PCA-2b cells, a fixation step was carried out: 80 μL of 10% formaldehyde was added to the cells, followed by a 20-minute incubation at room temperature before washing. The remaining cells were washed once with PBS. Subsequently, neutral red dye was extracted from each well using an acidified ethanol solution (1% acetic acid and 50% ethanol), and the absorbance at 540 nm was recorded using a BioTek plate reader. Both CTA (26) and neutral red plates were stained with 0.5% crystal violet in 20% methanol (27). After capturing the images, crystal violet was extracted with 10% glacial acetic acid and analyzed for absorbance at 590 nm using a BioTek plate reader. IC_50_ values were computed using the online AAT Biorequest IC_50_ Calculator (https://www.aatbio.com/tools/ic50-calculator). IC_50_ values were averaged between the assays and are shown as a range in the figures, and the average was presented as the IC_50_ value.

### RNA-seq analysis

Raw .fastq files from paired-end Illumina RNA-seq experiments on ACRJ-PC28 (18), DU-145, MDA-PCA-2b, and PC-3 (28) grown under standard conditions were downloaded from the European Nucleotide Archive (ENA; 29). FastQC (Galaxy version 0.74+ galaxy0; ref. 30) was used to check the quality of the data, and no trimming was required. Transcript and gene expression were quantified using Salmon quant (Galaxy version 1.10.1+ galaxy2; ref. 31) in quasi-mapping mode using the latest version of the human reference transcriptome assembly (32). DESeq2 (Galaxy version 2.11.40.8+ galaxy0; ref. 33) was used to determine differentially expressed genes (DEG) based on Salmon quant output. Genes with a significant adjusted *P* value < 0.05 and absolute [log_2_ (fold change)] > 1 were considered differentially expressed. DESeq2 generated a plot of the first two dimensions from a principal component analysis (PCA), run on the normalized counts of the samples. Finally, Gene Ontology and Kyoto Encyclopedia of Genes and Genomes (KEGG) pathway analyses were conducted using GoSeq (Galaxy version 1.50.0+ galaxy0; ref. 34). The results were visualized using PathView (Galaxy version 1.34.0+ galaxy0; ref. 35). Data were uploaded to the Galaxy web platform, and the public server at usegalaxy.org was used for analysis (36).

### Statistical analysis and IC_50_ value determination

IC_50_ values were computed using the online AAT Biorequest IC_50_ Calculator (https://www.aatbio.com/tools/ic50-calculator), which fits dose–response data to determine the concentration at which 50% cell viability inhibition occurs. For each cell line and drug combination, we generated multiple IC_50_ values from different cell viability assays (CTA, neutral red, and crystal violet) across 2 to 3 independent experiments. These values were averaged to provide a comprehensive assessment of drug sensitivity, with ranges reported to demonstrate the consistency across different assay methodologies. Drug potency was classified based on the following criteria: IC_50_ values below 1 μmol/L were considered potent, between 1 and 10 μmol/L as moderate, and above 10 μmol/L as weak, in accordance with established standards for anticancer compounds (37). Differential drug sensitivity between cell lines from Black and White donors was evaluated by comparing the magnitude of difference in IC_50_ values, with multifold differences (>3-fold) considered biologically significant. For RNA-seq analysis, statistical significance of DEGs was determined using DESeq2 with Benjamini–Hochberg correction for multiple testing. Genes with significant adjusted *P* value < 0.05 and absolute log_2_ (fold change) >1 were considered differentially expressed. PCA was performed on normalized counts to visualize global gene expression patterns and assess separation based on cell line derivation. Gene Ontology and KEGG pathway enrichment analyses were conducted using GoSeq to identify biological pathways associated with DEGs. All RNA-seq analyses were performed using the Galaxy web platform at usegalaxy.org.

## Results

**Figure 1.**
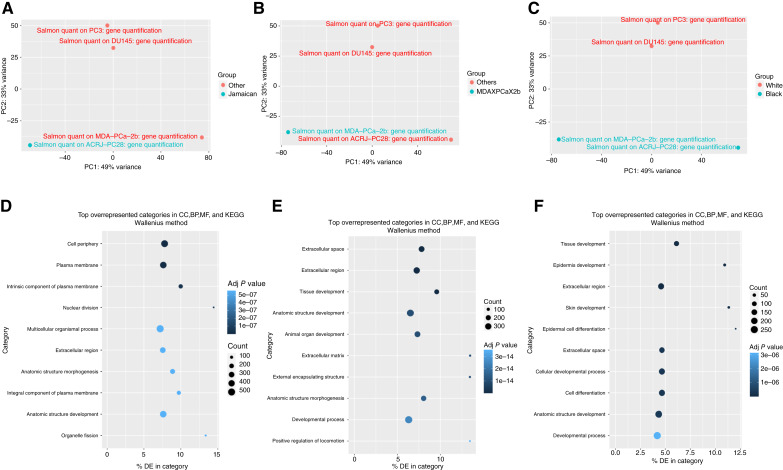
PCA and Gene Ontology analysis on Black- vs. White-derived prostate cancer (PCa) cell lines. **A,** PCA showing global gene expression of the Jamaican PCa cell line (ACRJ-PC28) vs. other PCa cell lines (DU-145, PC-3, and MDA-PCA-2b). **B,** PCA showing global gene expression of the Black-derived PCa cell line (MDA-PCA-2b) vs. other PCa cell lines (DU-145, PC-3, and ACRJ-PC28). **C,** PCA showing global gene expression of Black-derived PCa cell lines (ACRJ-PC28 and MDA-PCA-2b) vs. White PCa cell lines (DU-145 and PC-3). **D,** Top 10 overexpressed Gene Ontology terms from a comparison of ACRJ-PC28 vs. all other PCa cell lines (DU-145, PC-3, and MDA-PCA-2b). **E,** Top 10 overexpressed Gene Ontology terms from a comparison of the Black-derived PCa cell line (MDA-PCA-2b) vs. other PCa cell lines (DU-145, PC-3, and ACRJ-PC28). **F,** Top 10 overexpressed Gene Ontology terms from a comparison of the Black-derived PCa cell lines (ACRJ-PC28 and MDA-PCA-2b) vs. White PCa cell lines (DU-145 and PC-3). The *x*-axis is the percentage of genes in the category that have been identified as differentially expressed (DE). In the figure genes are clus. BP, biological process; CC, cellular component; MF, molecular function.

RNA-seq analysis demonstrated clear separation of cell lines based on ancestry origin through PCA (Fig. 1A–C). Raw .fastq files from paired-end Illumina RNA-seq experiments on ACRJ-PC28 (ENA accession: PRJEB45384; ref. 18), DU-145, MDA-PCA-2b, and PC-3 (28) grown under standard conditions were downloaded from the ENA (29). This ethnic-based clustering confirms the biological relevance of comparing drug responses between these models. Three key comparisons revealed substantial transcriptomic differences: (i) ACRJ-PC28 versus all other prostate cancer cell lines yielded 1,396 DEGs (1,123 underexpressed and 273 overexpressed); (ii) MDA-PCA-2b versus all other cell lines produced 887 DEGs (628 underexpressed and 259 overexpressed); and (iii) prostate cancer cell lines from Black versus White donors identified 633 DEGs (301 underexpressed and 332 overexpressed). Gene Ontology analysis (Fig. 1D–F) revealed enrichment of cancer-relevant pathways that may influence drug responsiveness, including cell adhesion, extracellular matrix organization, and signal transduction. The magnitude of transcriptomic differences between cell lines from Black and White donors (633 DEGs) underscores the molecular basis for potential ancestry-dependent drug sensitivities explored in subsequent cytotoxicity assessments.

FastQC (Galaxy version 0.74+ galaxy0; RRID: SCR_014583; ref. 30) was used to check the quality of the data, and no trimming was required. Transcript and gene expression were quantified using Salmon quant (Galaxy version 1.10.1+ galaxy2; RRID: SCR_017036; ref. 31) in quasi-mapping mode using the latest version of the human reference transcriptome assembly (32). DESeq2 (Galaxy version 2.11.40.8+ galaxy0; RRID: SCR_015687; ref. 33) was used to determine DEGs based on Salmon quant output. Gene Ontology and KEGG pathway analyses were conducted using GoSeq (Galaxy version 1.50.0+ galaxy0; RRID: SCR_017344; ref. 34). The results were visualized using PathView (Galaxy version 1.34.0+ galaxy0; RRID: SCR_017083; ref. 35). Data were uploaded to the Galaxy web platform (RRID: SCR_006281), and the public server at usegalaxy.org was used for analysis (36).

**Figure 2.**
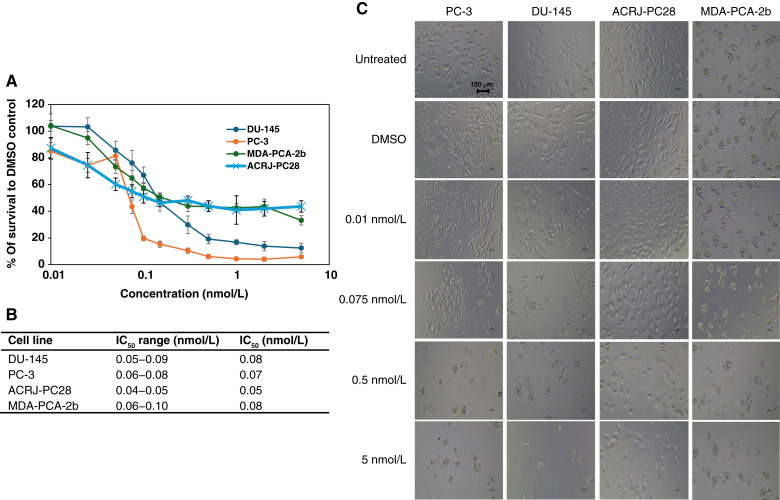
The cytotoxicity of docetaxel against prostate cancer (PCa) cell lines from Black and White patients. **A,** IC_50_ curves for all cell lines indicate comparable and very potent impact on all the cell lines. **B,** Table of the IC_50_ range and average IC_50_ value for each cell line. **C,** Microscopy at ×20 magnification for each cell line in the presence (at increasing concentrations) and absence of docetaxel. The IC_50_ range was calculated using data from two crystal violet assays, one CTA assay, and one neutral red assay for all the PCa cell lines. Average IC_50_ values were calculated using all values in the IC_50_ range for each PCa cell line.

Among the five anticancer drugs evaluated, docetaxel exhibited exceptional potency across all four cell lines, with IC_50_ values ranging from 0.05 to 0.08 nmol/L (Fig. 2). Despite this narrow range, ACRJ-PC28 showed modestly enhanced sensitivity (IC_50_ = 0.05 nmol/L) compared with MDA-PCA-2b and DU-145 (both IC_50_ = 0.08 nmol/L), representing a 1.6-fold difference. ACRJ-PC28 was the only cell line used in this study that did not express the AR [Valentine and colleagues (18)]. Among the DEGs, *AR* was among the top 10 underexpressed genes in ACRJ-PC28, which could account for its lack of protein expression. In contrast, there was evidence of *AR* expression in all other cell lines (38). *TNFRSF14* and *C1QTNF9B* were also among the genes underexpressed in ACRJ-PC28. Both genes encode members of the TNF protein family. Another member of the TNF family that was overexpressed was *TNFSF11* (*RANKL*). *TNFSF11* activates the antiapoptotic kinase AKT/PKB through a signal transduction pathway that involves TRAF. Interestingly, *TRAF* was among the underexpressed genes (Supplementary Table S1).

**Figure 3.**
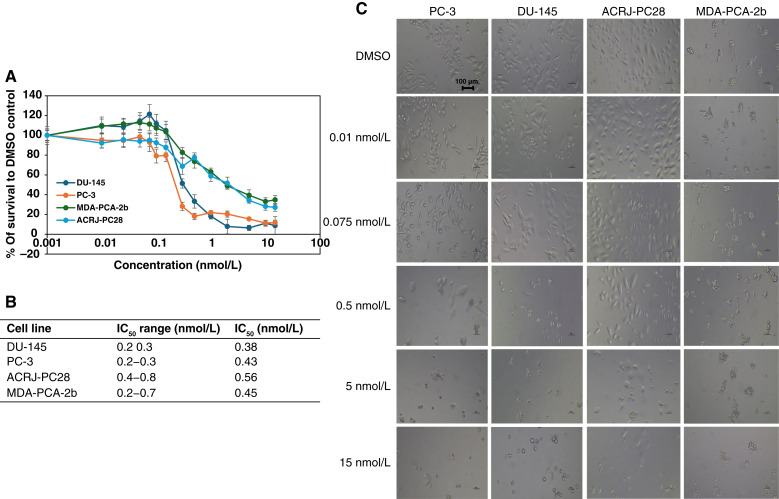
The cytotoxicity of cabazitaxel against prostate cancer cell lines from Black and White patients. **A,** IC_50_ curves for all cell lines indicate comparable and potent impact on all the cell lines. **B,** Table of the IC_50_ range and average IC_50_ value for each cell line. **C,** Microscopy at ×20 magnification for each cell line in the presence (at increasing concentrations) and absence of cabazitaxel. The IC_50_ range was calculated using data from two crystal violet assays and one neutral red assay for PC-3, DU-145, and ACRJ-PC28 cell lines. For the MDA-PCA-2b cell line, the IC_50_ range was determined from two crystal violet assays and one CTA assay. Average IC_50_ values were calculated using all values in the IC_50_ range for each prostate cancer cell line.

Similarly, cabazitaxel demonstrated potent activity across all cell lines (IC_50_ range, 0.38–0.56 nmol/L) without clear ancestry-dependent patterns (Fig. 3). DU-145 (White ancestry) showed the highest sensitivity (IC_50_ = 0.38 nmol/L), whereas ACRJ-PC28 (Black ancestry) exhibited the lowest (IC_50_ = 0.56 nmol/L), though this 1.47-fold difference remains relatively modest. *MDR1* was neither under nor overexpressed in prostate cancer cell lines from Black donors. Notably, both taxanes maintained efficacy regardless of *AR* expression status, highlighting their value in diverse prostate cancer phenotypes. In contrast to taxanes, androgen-targeted therapies exhibited striking ancestry-dependent patterns.

**Figure 4.**
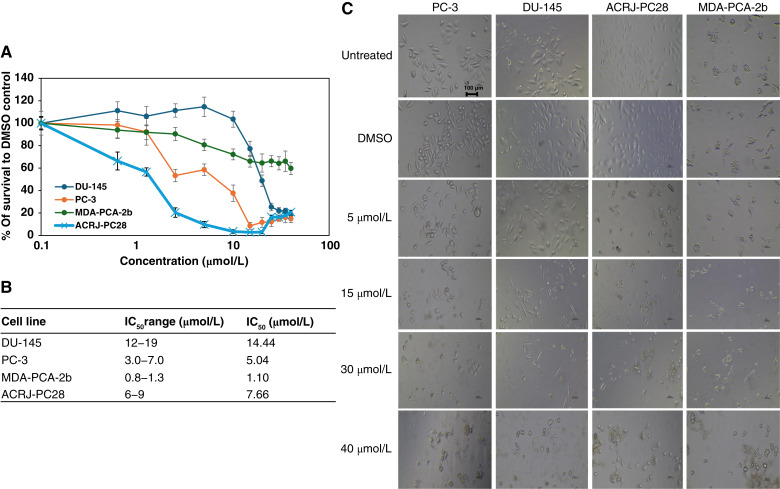
Showing the cytotoxicity of abiraterone prostate cancer cell lines from Black and White patients. **A,** IC_50_ curves for all cell lines indicate greatest impact on the Black-derived ACRJ-PC28 cell line. **B,** Table of the IC_50_ range and average IC_50_ value for each cell line. **C,** Microscopy at ×20 magnification for each cell line in the presence (at increasing concentrations) and absence of abiraterone. The IC_50_ range was calculated using data from two crystal violet assays and one neutral red assay for PC-3, DU-145, and ACRJ-PC28 cell lines. For the MDA-PCA-2b cell line, the IC_50_ range was determined from one crystal violet assay, one neutral red assay, and one CTA assay. Average IC_50_ values were calculated using all values in the IC_50_ range for each prostate cancer cell line.

Abiraterone demonstrated remarkable selectivity toward ACRJ-PC28 (IC_50_ = 1.10 μmol/L), being 4.6-fold more potent than against PC-3 (IC_50_ = 5.04 μmol/L), 7.0-fold more potent than against MDA-PCA-2b (IC_50_ = 7.66 μmol/L), and 13.1-fold more potent than against DU-145 (IC_50_ = 14.44 μmol/L; Fig. 4). This preferential efficacy against the Black-derived ACRJ-PC28 line was observed despite significant underexpression of *AR* and *PSA* compared with other cell lines. The moderate sensitivity of MDA-PCA-2b (IC_50_ = 7.66 μmol/L) was observed despite its *AR* overexpression. The weakest impact of Abiraterone was observed in the White prostate cancer cell line, DU-145. *CYP17* was not observed among the DEGs in MDA-PCA-2b compared with the other prostate cancer cell lines.

**Figure 5.**
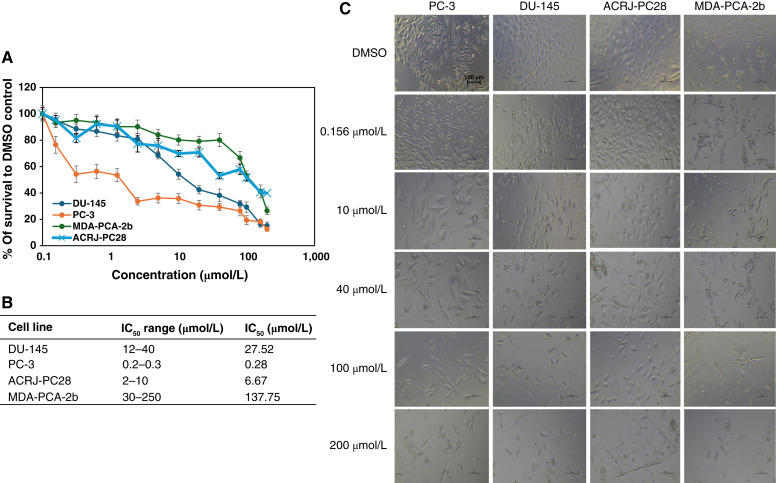
Showing the cytotoxicity of olaparib against prostate cancer cell lines from Black and White patients. **A,** IC_50_ curves for all cell lines indicate moderate sensitivity on the Black-derived ACRJ-PC28 cell line. **B,** Table of the IC_50_ range and average IC_50_ value for each cell line. **C,** Microscopy at ×20 magnification for each cell line in the presence (at increasing concentrations) and absence of olaparib. The IC_50_ range was calculated using data from two crystal violet assays and one neutral red assay for PC-3 and DU-145 cell lines. For the MDA-PCA-2b and ACRJ-PC28 cell lines, the IC_50_ range was determined from two crystal violet assays and one CTA assay. Average IC_50_ values were calculated using all values in the IC_50_ range for each prostate cancer cell line.

Olaparib demonstrated variable efficacy across cell lines without clear ancestry-dependent patterns (Fig. 5). PC-3 (White ancestry) showed exceptional sensitivity (IC_50_ = 0.28 μmol/L), whereas MDA-PCA-2b (Black ancestry) exhibited profound resistance (IC_50_ = 137.75 μmol/L), representing a 492-fold difference. ACRJ-PC28 demonstrated moderate sensitivity (IC_50_ = 6.67 μmol/L). *PARP* was found to be the least underexpressed gene in our ACRJ-PC28 cell line, with *BRCA1/2* also showing underexpression. Interestingly, *RAD51* which plays a role in repairing double-stranded DNA breaks, was also underexpressed in our cell line. *FGFR* was overexpressed in ACRJ-PC28 cells compared with other cell lines (Supplementary Table S1). In contrast to ACRJ-PC28, *PARP* and *BRCA1/2* were neither underexpressed nor overexpressed in the MDA-PCA-2b cell line; however, *TIPARP* which codes for a member of the PARP superfamily, was underexpressed (Supplementary Table S2). Other potential targets for olaparib, including *VEGFC* and *FGF2* in addition to members of the EGF family (*EGFLAM*, *HBEGF*, and *EGFL7*), were also underexpressed in this cell line.

**Figure 6.**
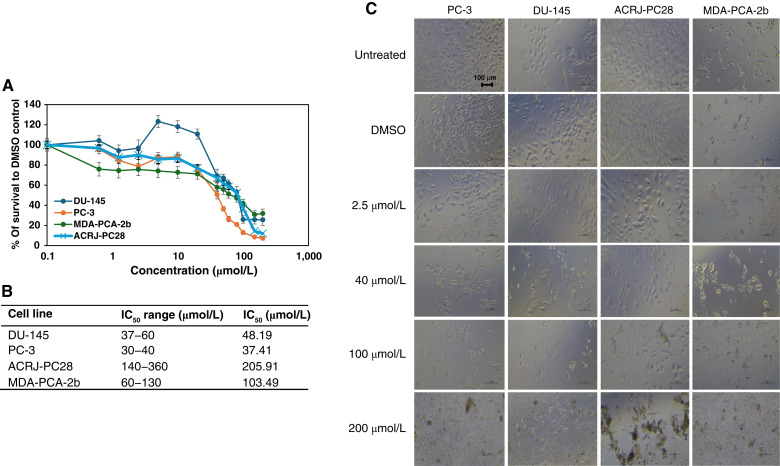
Showing the cytotoxicity of enzalutamide against prostate cancer cell lines from Black and White patients. **A,** IC_50_ curves for all cell lines indicate least impact on the Black-derived ACRJ-PC28 cell line. **B,** Table of the IC_50_ range and average IC_50_ value for each cell line. **C,** Microscopy at ×20 magnification for each cell line in the presence (at increasing concentrations) and absence of enzalutamide. The IC_50_ range was calculated using data from two crystal violet assays and one neutral red assay for MDA-PCA-2b cell line. For the remaining cell lines (ACRJ-PC28, DU-145, and PC-3), the IC_50_ range was determined from two crystal violet assays, one neutral red assay, and one CTA assay. Average IC_50_ values were calculated using all values in the IC_50_ range for each prostate cancer cell line.

Among the anticancer drugs evaluated, enzalutamide (Fig. 6) exhibited the most pronounced ancestry-dependent response pattern, being the weakest at reducing the viability of all cell lines. Black-derived cell lines showed substantially reduced sensitivity, with ACRJ-PC28 (IC_50_ = 205.91 μmol/L) and MDA-PCA-2b (IC_50_ = 103.49 μmol/L) being 5.5-fold and 2.8-fold less responsive, respectively, than White-derived PC-3 cells (IC_50_ = 37.41 μmol/L). This consistent ancestry-associated resistance pattern across both Black-derived cell lines suggests fundamental biological differences in enzalutamide response mechanisms between ancestry groups. Interestingly, whereas the MDA-PCA-2b cell line showed low sensitivity to enzalutamide, it exhibited greater sensitivity to abiraterone than to enzalutamide, despite its overexpression of *AR* (Supplementary Table S2).

**Figure 7.**
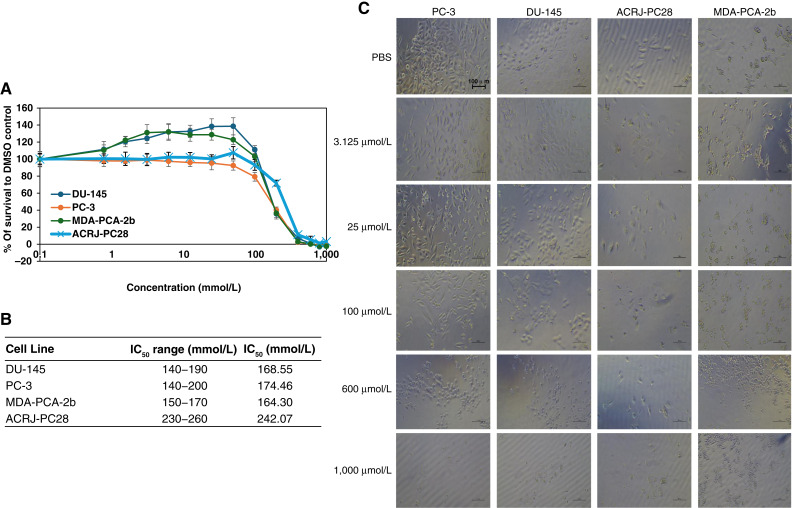
The cytotoxicity of betaine against prostate cancer cell lines from Black and White patients. **A,** IC_50_ curves for all cell lines indicate weak impact on all the cell lines. **B,** Table of the IC_50_ range and average IC_50_ value for each cell line. **C,** Microscopy at ×20 magnification for each cell line in the presence (at increasing concentrations) and absence of betaine. The IC_50_ range was calculated using data from two crystal violet assays and one neutral red assay for PC-3, DU-145, and MDA-PCA-2b cell lines. For the ACRJ-PC28 cell line, the IC_50_ range was determined from two crystal violet assays and one neutral red assay. Average IC_50_ values were calculated using all values in the IC_50_ range for each prostate cancer cell line.

The natural product betaine (Fig. 7) demonstrated the least impact on all cell lines. Cytotoxicity ranged between 164.3 and 242.07 mmol/L, and the impact was least seen in ACRJ-PC28 than in all other cell lines that were comparatively affected. It was the only product that dissociated the clusters of MDA-PCA-2b cells when killed at 600 and 1,000 mmol/L (Fig. 6).

The ACRJ-PC28 cell line was developed from a patient of Afro-Caribbean ancestry who presented with a PSA of 441.2 ng/mL and nodules in the right lobe of the prostate (cT2b). Biopsy confirmed adenocarcinoma with a Gleason score of 4 + 4. The patient was immediately placed on ADT, bicalutamide. On the subsequent dose of bicalutamide, the patient’s therapy included hormone therapy and lectrum. The patient’s staging bone scan revealed multiple bony lesions in the skull, sternum, scapulae, ribs, multiple levels of the thoracic–lumbar vertebrae, and pelvis. The patient died a little more than a year later. The patient’s biopsy specimen revealed the presence of AMACR, *AR*, NKX3.1, and PSA, but the protein expression for all these was lost in the cell line (18). Our characterizations revealed that ACRJ-PC28 is a prostate cancer with a neuroendocrine phenotype that tends to be aggressive, as observed in this patient.

The whole-genome sequencing analysis did not detect any exonic mutations in *AR* in the ACRJ-PC28 cell line; therefore, the initial course of treatment was bicalutamide (a nonsteroidal antiandrogen) followed by lectrum, which suppresses gonadotrope secretion of luteinizing hormone. A preliminary pathology report stated that *AR* is expressed in 90% of tissues. From our cytotoxic data, both docetaxel and abiraterone, which are typically paired in a clinical setting, were most effective in our Black-derived ACRJ-PC28 cell line.

## Discussion

Our study reveals significant differences in gene expression profiles and drug responses between prostate cancer cell lines derived from Black and White patients, highlighting the importance of ancestry-specific approaches to preclinical drug evaluation. The ability of RNA-seq analysis to clearly separate cell lines by ancestry through PCA confirms the biological relevance of our comparative approach and suggests fundamental molecular differences that may influence therapeutic outcomes.

The taxane compounds, docetaxel and cabazitaxel, demonstrated potent nanomolar activity across all cell lines regardless of ancestry, though with subtle variations. The ability of docetaxel to bind to tubulin prevents cellular division, but research has also identified an additional mechanism involving inhibition of TNFα-induced NF-κB, which is known to suppress apoptosis and has been linked to docetaxel chemoresistance (39). The heightened response observed in ACRJ-PC28, along with its DEG profile, suggests the potential therapeutic advantages of the drug in patients with similar molecular profiles. There is also evidence of increased overall survival of patients with prostate cancer on docetaxel when paired with an *AR* inhibitor such as abiraterone (39).

Similar to docetaxel, cabazitaxel is also an inhibitor of microtubular depolarization (40). However, cabazitaxel is primarily used as second-line therapy for docetaxel-resistant metastatic castration-resistant prostate cancer (mCRPC; 12). Cabazitaxel was developed to ensure poor affinity for P-glycoprotein, addressing a common mechanism of docetaxel resistance associated with increased expression of the multidrug resistance (*MDR*) 1 gene, which encodes P-glycoprotein. This property makes cabazitaxel potentially more effective in improving overall survival and quality of life in resistant cases (41). The efficacy of these taxanes regardless of *AR* status suggests they remain valuable options for diverse prostate cancer phenotypes, including *AR*-negative neuroendocrine variants like ACRJ-PC28.

In striking contrast to the taxanes, androgen-targeted therapies exhibited pronounced ancestry-dependent response patterns. Abiraterone’s remarkable selectivity toward ACRJ-PC28 aligns with clinical observations from George and colleagues (42), who reported enhanced abiraterone response in Black patients with mCRPC. Androgen deprivation is a common approach to managing advanced prostate cancer. Even at low testosterone levels induced by castration, the presence of androgens from alternative sources like adrenal glands and prostate cancer tissue can sustain tumor growth. Abiraterone functions by inhibiting *CYP17*, an enzyme crucial for cortisol and androgen synthesis (14).

The paradoxical exceptional sensitivity of ACRJ-PC28 to abiraterone despite significant *AR* underexpression represents a particularly intriguing finding. This suggests that alternative molecular mechanisms beyond canonical *AR* signaling may mediate abiraterone’s enhanced efficacy in some Black patients (42), postulated that tissues in Black men might be more susceptible to abiraterone’s effects on adrenal glands. Although PC-3 and DU-145 cell lines reportedly express *AR* mRNA and protein, conflicting studies suggest their absence in some contexts (43), highlighting the complex and heterogeneous nature of *AR* signaling in prostate cancer.

The profound differences in olaparib efficacy across cell lines seem driven by cell-specific molecular characteristics rather than ancestry alone. Olaparib inhibits PARP, impeding single-stranded DNA break repair and demonstrating antiangiogenic effects (44). Its targets beyond PARP include growth factors crucial for prostate cancer progression, including VEGF, FGF2, TGFB1, EGF, and IGF1 (45, 46). The exceptional sensitivity of PC-3 cells aligns with findings by Sargazi and colleagues (45), who correlated this response with biallelic mutations in *PTEN* and *BRCA1*. PARP inhibitors demonstrate synthetic lethality in cells with *BRCA1/2* deficiencies (47).

The moderate sensitivity of ACRJ-PC28 to olaparib despite *PARP1* underexpression may be explained by its concurrent underexpression of DNA repair genes like *RAD51* and overexpression of *FGFR*, which induces osteogenesis and angiogenesis (48). Conversely, the weaker response in DU-145 may relate to abundant *PTEN* expression (49), whereas MDA-PCA-2b’s profound resistance might stem from underexpression of *TIPARP*, *VEGFC*, *FGF2*, and EGF family members rather than direct PARP pathway alterations. These complex patterns underscore the need for comprehensive molecular profiling to guide PARP inhibitor therapy.

Enzalutamide exhibited the most pronounced ancestry-dependent response pattern among all tested compounds, with both Black-derived cell lines showing substantially reduced sensitivity compared with White-derived lines. Enzalutamide is an *AR* inhibitor that disrupts testosterone–cell interactions, nuclear translocation, DNA binding, and coactivator recruitment (15). The limited impact on ACRJ-PC28 cells aligns with their *AR*-negative status (18), whereas MDA-PCA-2b’s weak response despite *AR* overexpression likely stems from its androgen-independent nature and known *AR* mutations (50). The recent FDA approval of enzalutamide with leuprolide for nonmetastatic CRPC highlights evolving treatment paradigms (51), but our findings suggest potential limitations in Black patients.

It is particularly notable that MDA-PCA-2b showed greater sensitivity to abiraterone than enzalutamide despite *AR* overexpression, paralleling the clinical observation that Black men with mCRPC respond better to abiraterone than their White counterparts (42). This consistent ancestry-based pattern suggests fundamental biological differences in androgen signaling pathway response that warrant further investigation and may inform treatment selection.

Betaine demonstrated limited cytotoxicity across all cell lines, with minimal ancestry-dependent patterns. Previous studies have shown that betaine can suppress proliferation in DU-145 cells through oxidative stressmediated apoptosis and inflammation (52), inhibit angiogenesis via NF-κΒ and Akt pathway suppression (53), and promote osteogenesis in human osteoblastic cells (54). Although its *in vitro* potency is weak, betaine’s water solubility offers potential drug delivery advantages, and its unique ability to dissociate MDA-PCA-2b cell clusters suggests potential utility in combination therapies, warranting further investigation.

The ACRJ-PC28 cell line’s aggressive neuroendocrine phenotype mirrors the clinical course of its source patient, who succumbed within a year despite ADT. The loss of canonical prostate cancer markers (AMACR, *AR*, NKX3.1, and PSA) between biopsy and cell line establishment reflects the cellular evolution commonly observed in treatment-resistant disease. Hormone receptor loss can occur during *in vitro* growth conditions (55), though the original *AR* expression in 90% of tissue suggests either selection of a minor *AR*-negative population during establishment or epigenetic alterations over time. The whole-genome sequencing finding of no exonic *AR* mutations, coupled with pronounced sensitivity to both docetaxel and abiraterone, raises the question of whether this combination might have improved the patient’s outcome compared with the bicalutamide and lectrum regimen received.

The evolution of *AR* resulting in constitutively active variants lacking the ligand-binding domain represents a common route to ADT resistance (56). *AR* transcriptional activity can be altered through gene amplification or mutations causing inappropriate responses to antagonists like bicalutamide or enzalutamide (57). Given these resistance mechanisms, our findings support an aggressive approach to advanced prostate cancer with a framework for immediate treatment adjustments based on molecular profiles.

Among the genes of interest identified, *TNFRSF14* underexpression in both Black-derived prostate cancer cell lines represents a particularly intriguing finding warranting further investigation. This consistent alteration in TNF family signaling may contribute to the observed ancestry-dependent drug responses, potentially serving as both a biomarker and therapeutic target.

Our study has several limitations that should be acknowledged. Cell lines imperfectly mirror tumor heterogeneity, and responses may not directly translate to clinical outcomes. The number of cell lines examined (two Black-derived and two White-derived) provides important initial insights, but broader panels would strengthen conclusions about ancestry-dependent patterns. Additionally, the loss of *AR* expression in ACRJ-PC28 during establishment creates some uncertainty about how closely it represents the original tumor biology. Despite these limitations, the clear ancestry-dependent patterns observed, particularly for androgen-targeted therapies, provide compelling evidence for biological differences in drug response that merit clinical consideration.

The heterogeneity of prostate cancer necessitates diverse therapeutic approaches. Our findings demonstrate that drug selectivity patterns vary markedly by drug class and cellular context, with abiraterone and olaparib showing cell-specific selectivity and enzalutamide exhibiting clear ancestry-dependent effects, whereas taxanes maintain broad efficacy. These patterns underscore the importance of incorporating diverse prostate cancer models in preclinical drug screening to identify optimized therapeutic strategies for patients across ancestral backgrounds.

Clinical validation of these ancestry-dependent drug response patterns represents a critical next step. Future studies should include retrospective analysis of treatment outcomes in diverse patient cohorts, development of patient-derived xenograft (PDX) models from both Caribbean and African American populations, and prospective clinical trials stratified by genetic ancestry. Collaboration with existing biobanks and utilization of available NCI PDX models from African American patients could accelerate validation of these findings and translation to personalized treatment approaches.

### Conclusions

This study demonstrates that ancestry-specific drug responses in prostate cancer reflect fundamental biological differences at the molecular level. Our comprehensive analysis of the novel ACRJ-PC28 cell line alongside established models revealed three key patterns: (i) taxanes maintained efficacy across all cell lines regardless of ancestry or *AR* status; (ii) androgen-targeted therapies showed striking ancestry-dependent responses, with abiraterone demonstrating 4.6- to 13.1-fold greater potency in ACRJ-PC28 despite its *AR*-negative status; and (iii) enzalutamide consistently showed reduced efficacy in Black-derived cell lines. The paradoxical sensitivity of *AR*-negative ACRJ-PC28 to abiraterone provides mechanistic insights into clinical observations of enhanced response to this therapy in Black patients with mCRPC. The consistent underexpression of *TNFRSF14* and other TNF family genes in Black-derived prostate cancer cell lines highlights potential molecular mechanisms underlying these differential responses. These findings have immediate clinical implications, suggesting that Black patients with prostate cancer with aggressive disease might benefit more from abiraterone than enzalutamide, potentially in combination with taxanes. The ACRJ-PC28 model addresses a critical gap in prostate cancer research by providing the first Afro-Caribbean–derived cell line, enabling more precise preclinical evaluation of therapies for populations with disproportionate disease burden. Future research should focus on validating these ancestry-dependent response patterns in PDXs and clinical cohorts, exploring the mechanistic role of TNF family genes in drug response, and developing precision medicine approaches that optimize treatment selection based on molecular profiles beyond ancestry alone.

## Supplementary Material

Supplementary Table 1 DEGs in ACRJ-PC28 vs Other PCa. This table presents the complete list of 1396 differentially expressed genes (DEGs) identified when comparing the novel Afro-Caribbean ACRJ-PC28 cell line against all other PCa cell lines (DU-145, PC-3, and MDA-PCA-2b). For each gene, we provide the gene symbol, gene name, log2 fold change, adjusted p-value, and functional categorization. Key genes highlighted include AR, TNFRSF14, C1QTNF9B, TNFSF11, TRAF, PARP1, BRCA1/2, RAD51, and FGFR family members, which may contribute to the unique drug response profile observed in ACRJ-PC28 cells.

Supplementary Table 2 DEGs in MDA-PCA-2b vs Other PCa. This supplementary table presents the 887 differentially expressed genes identified when comparing the Black-derived MDA-PCA-2b cell line against all other PCa cell lines (ACRJ-PC28, DU-145, and PC-3). The table includes gene symbols, descriptions, log2 fold changes, adjusted p-values, and functional classifications. Notable genes include overexpressed AR and underexpressed TNFRSF14 and TIPARP, along with members of growth factor families (VEGFC, FGF2, EGFLAM, HBEGF, and EGFL7) that may influence the cell line's unique response to targeted therapies, particularly its resistance to Olaparib and reduced sensitivity to Enzalutamide.

Supplementary Table 3 DEGs: Black-derived vs White-derived PCa Lines. This table documents the 633 genes differentially expressed between Black-derived (ACRJ-PC28 and MDA-PCA-2b) and White-derived (DU-145 and PC-3) PCa cell lines. The data includes gene identifiers, annotations, expression fold changes, statistical significance values, and functional pathway associations. The table highlights genes consistently underexpressed in Black-derived cell lines, including TNFRSF14 and other genes involved in androgen signaling, DNA damage repair, and growth factor pathways that may contribute to the observed ancestry-dependent differences in drug sensitivity, particularly to androgen-targeted therapies and PARP inhibitors.

## Data Availability

RNA-seq data for ACRJ-PC28 are available from the ENA under accession number PRJEB45384. RNA-seq data for DU-145, MDA-PCA-2b, and PC-3 cell lines are available under accession numbers previously published (28). All other data supporting the findings of this study, including IC_50_ values, statistical analyses, and gene expression data, are available within the article and its supplementary materials (Supplementary Tables S1–S3). The ACRJ-PC28 cell line is available from the corresponding author upon reasonable request and completion of a material transfer agreement.
